# An unusual case of chronic eyelid infection associated with *Staphylococcus aureus* and *Morganella morganii*: A case report

**DOI:** 10.1097/MD.0000000000051471

**Published:** 2026-10-30

**Authors:** Ju-Wen Yang, Liang-Che Chang

**Affiliations:** a Department of Ophthalmology, Chang Gung Memorial Hospital, Keelung, Taiwan; b College of Medicine, Chang Gung University, Kwei-shan, Taoyuan, Taiwan; c Department of Anatomic Pathology, Chang Gung Memorial Hospital, Keelung, Taiwan.

**Keywords:** conjunctiva, eyelids, methicillin-resistant *Staphylococcus aureus*, *Morganella morganii*

## Abstract

**Rationale:**

*Morganella morganii* is an uncommon opportunistic pathogen, and eyelid infections attributable to this organism are exceptionally rare.

**Patient concerns:**

A 53-year-old male presented with persistent discharge and progressive structural changes of the left eyelid.

**Diagnoses:**

Microbiological analysis of conjunctival discharge initially revealed oxacillin-susceptible *Staphylococcus aureus* and *M morganii*. Intraoperative cultures subsequently yielded methicillin-resistant *S aureus* (MRSA) and *M morganii*. Histopathological examination demonstrated acute and chronic inflammatory changes with fistulous tract formation.

**Interventions:**

Initial empirical treatment included topical levofloxacin and tobramycin ophthalmic ointment, together with a brief course of oral amoxicillin/clavulanate. Based on subsequent culture and antimicrobial susceptibility findings, topical vancomycin was added to levofloxacin for 2 months, followed by intermittent topical antimicrobial therapy adjusted according to the clinical course. As conjunctival fistulae and chronic inflammation persisted despite extended medical treatment, surgical excision, limited debridement, and conjunctival wound closure were subsequently performed.

**Outcomes:**

Despite initial medical management, the patient developed multiple palpebral conjunctival openings and persistent fistulae over a 2-year period, with the discharge changing from purulent yellow secretions to thick, toothpaste-like material. Histopathological examination revealed acute and chronic inflammation with fistulous tract formation. Following surgical intervention, marked improvement was noted, including regression of the eyelid mass, closure of the fistulae, and resolution of discharge. At 1.5 years of postoperative follow-up, the eyelid remained stable without recurrence of fistulae or discharge.

**Lessons:**

This case highlights a chronic polymicrobial eyelid and conjunctival infection involving *S aureus* and *M morganii*, with methicillin-resistant *S aureus* isolated at the time of surgery. The findings suggest that *M morganii* may contribute to sustaining chronic periocular infection in susceptible hosts. In this patient, persistent infection complicated by fistula formation ultimately required surgical management after prolonged antimicrobial therapy failed to achieve resolution.

## 1. Introduction

*Morganella morganii* is an uncommon opportunistic gram-negative bacillus that is rarely implicated in ocular or periocular infections. Large microbiological studies report its prevalence in bacterial keratitis to be approximately 1%,^[1]^ and only isolated case reports have described ocular adnexal involvement, such as orbital abscess extension from dacryocystitis.^[2]^ Its capacity to acquire multidrug-resistant genes, together with growing evidence of its pathogenic potential,^[3]^ highlights the importance of early organism identification and targeted antimicrobial therapy to prevent chronic or recurrent disease.

In contrast, *Staphylococcus aureus*, particularly methicillin-resistant strains (methicillin-resistant *S aureus* [MRSA]), is a major cause of severe periocular and orbital infections.^[4–6]^ Recent reports have demonstrated that MRSA-associated orbital cellulitis may present atypically, frequently without preceding sinusitis, and may require early culture-guided antimicrobial therapy with timely surgical drainage when abscesses or necrotic complications arise.^[5,6]^ These observations highlight the importance of considering resistant or uncommon pathogens in patients with persistent, refractory, or atypical eyelid and orbital infections. This case illustrates the clinical challenges of a chronic palpebral conjunctival infection characterized by progressive fistula formation and polymicrobial cultures involving *S aureus* and *M morganii*.

## 2. Case presentation

The patient was a 53-year-old male with diabetes mellitus and end-stage renal disease who presented with persistent left ocular discharge. His diabetes was well-controlled with glimepiride 1 mg daily, with HbA1c levels generally < 7%. He had been receiving maintenance hemodialysis for approximately 7 years and had no known history of immunodeficiency, recurrent infections, immunosuppressive therapy, or other medications known to significantly compromise immune function. His ocular history was notable for proliferative diabetic retinopathy, treated with bilateral trans–pars plana vitrectomy for tractional retinal detachment 8 years earlier. At presentation, the ocular surface findings and discharge were consistent with bacterial conjunctivitis (Fig. 1). Empirical treatment was initiated with topical levofloxacin 4 times daily, tobramycin ophthalmic ointment at bedtime, and oral amoxicillin/clavulanate for 1 week. Conjunctival discharge cultures subsequently detected *M morganii* and oxacillin-susceptible *S aureus*, the former being an uncommon gram-negative opportunistic pathogen rarely associated with ocular or periocular infections. Antimicrobial susceptibility testing revealed susceptibility of *M morganii* to levofloxacin and of *S aureus* to vancomycin and oxacillin (Table 1).

**Table 1:** Microbiological culture findings and antimicrobial susceptibility profiles of conjunctival discharge obtained at initial presentation and at the time of surgery.

Antimicrobial agent	Initial culture		Culture at surgery		
	*M morganii* (Light)	*S aureus* (Moderate)	MRSA (Moderate)	*M morganii* isolate 1 (moderate)	*M morganii* isolate 2 (moderate)
Amikacin	S	-	-	S	R
Ceftazidime	S	-	-	S	S
Clindamycin	-	R	R	-	-
Ciprofloxacin	S	-	-	S	S
Cefoperazone/sulbactam	S	-	-	S	S
Ceftriaxone	S	-	-	S	S
Cefuroxime	R	-	-	R	R
Cefazolin	R	-	-	R	R
Erythromycin	-	R	R	-	-
Ertapenem	S	-	-	S	S
Fusidic acid	-	S	-	-	-
Gentamicin	S	-	-	S	I
Levofloxacin	S	-	-	S	S
Oxacillin	-	S	R	-	-
Penicillin	-	R	R	-	-
Ampicillin–sulbactam	S	-	-	I	S
Trimethoprim–sulfamethoxazole	-	S	S	-	-
Teicoplanin	-	S	S	-	-
Tigecycline	-	S	S	-	-
Piperacillin–tazobactam	S	-	-	S	S
Vancomycin	-	S	S	-	-

Two *M morganii* isolates were reported from the same intraoperative specimen with different susceptibility profiles; further strain characterization and molecular typing were not performed.

- means not tested or not reported.

I = intermediate, R = resistant, MRSA = methicillin-resistant *Staphylococcus aureus*, S = susceptible.

**Figure 1.**
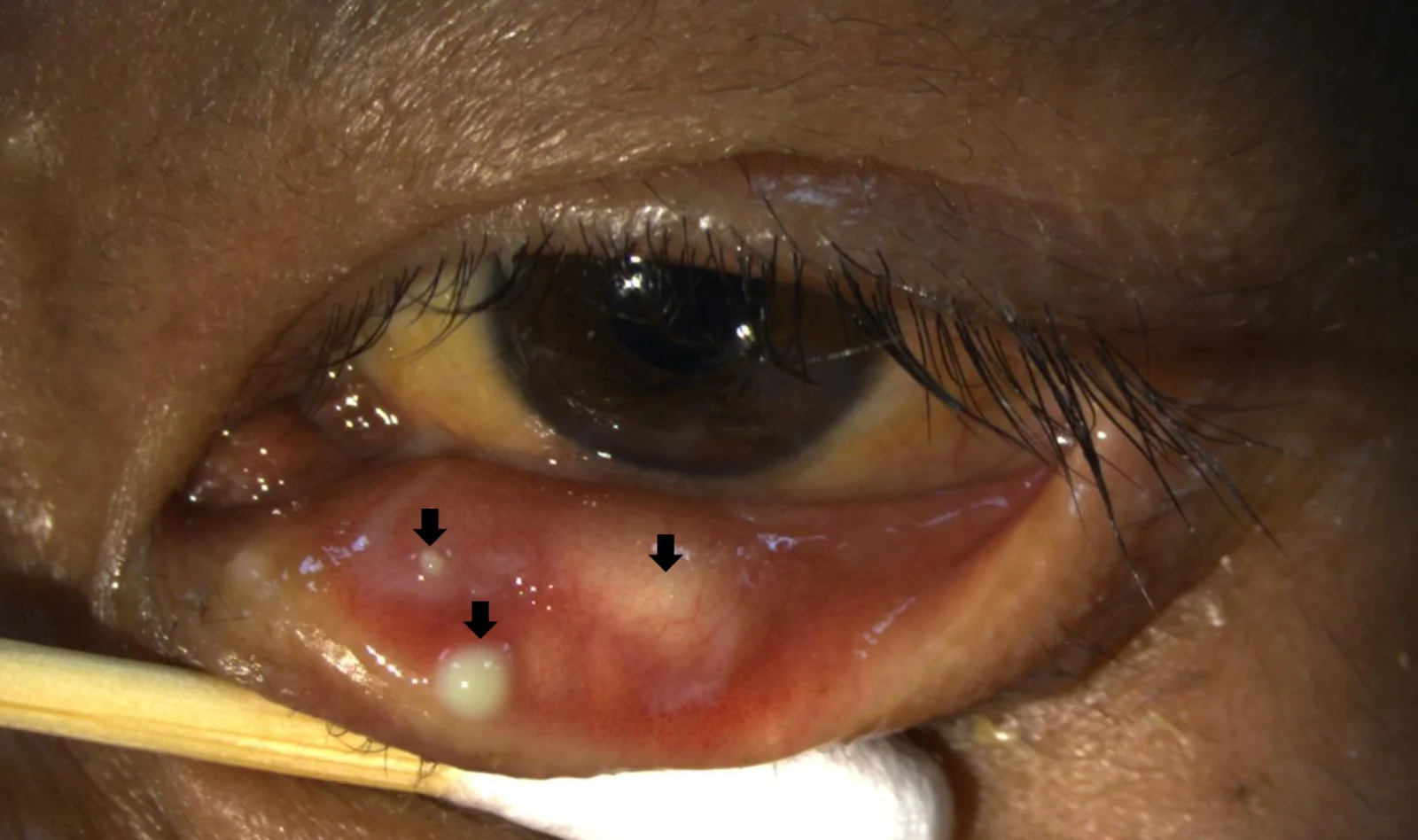
Clinical photograph of the left eye at initial presentation showing pustular lesions on the lower palpebral conjunctiva (arrows), associated with persistent conjunctival discharge.

Based on the antimicrobial susceptibility results, topical vancomycin 25 mg/mL 4 times daily was added to topical levofloxacin and continued for 2 months. Levofloxacin was subsequently continued as monotherapy, with clinical improvement noted approximately 8 months after the initial presentation. Topical therapy was then changed to chloramphenicol twice daily for 8 months. Following clinical improvement, topical antimicrobial therapy was discontinued for 3 months. When conjunctival discharge recurred, topical sulfamethoxazole 4% ophthalmic solution twice daily was prescribed for 3 months. After a subsequent recurrence, topical levofloxacin was resumed for an additional 2 months. The clinical course, microbiological findings, treatment, and outcomes are presented in Table 2. Despite regular follow-up and patient-reported adherence to the prescribed topical antimicrobial therapy, the condition gradually worsened. Approximately 8 months after the initial presentation, multiple punctate openings appeared on the lower palpebral conjunctiva, accompanied by changes in the discharge character. Initially yellow and purulent, the discharge gradually became more viscous, ultimately assuming a whitish, toothpaste-like appearance, raising concern for evolving fistulous tract formation.

**Table 2:** Timeline of the patient’s clinical course, including microbiological findings, antimicrobial treatment, progression of conjunctival fistulae, surgical intervention, and postoperative outcome. Time points are referenced from the initial presentation unless otherwise specified.

Initial presentation Empirical topical levofloxacin and tobramycin ophthalmic ointment + oral amoxicillin/clavulanate
↓
Initial culture *S aureus* + *M morganii* → topical vancomycin (25 mg/mL) × 2 months + continued topical levofloxacin
↓
8 months after initial presentation Multiple conjunctival openings + whitish, toothpaste-like discharge
↓
2 years after initial presentation Persistent conjunctival fistulae + thickened, indurated eyelid
↓
Surgery Excision of involved/fistulous tissue + limited debridement + conjunctival wound closure
↓
Culture/Pathology MRSA + *M morganii* + acute and chronic inflammation with fistulous tract formation
↓
Outcome (1.5 years after surgery) Resolution of eyelid mass + fistula closure + no further discharge

MRSA = methicillin-resistant *Staphylococcus aureus*.

Two years after the initial presentation, the left lower palpebral conjunctiva developed persistent fistulous tracts connecting the previously identified openings (Fig. 2). Concurrently, the upper eyelid became progressively thickened, indurated, and firm on palpation. Although acute pain was absent, the patient reported persistent fullness and localized eyelid discomfort, indicating ongoing inflammation and tissue remodeling. Given the chronic course and fistula formation, alternative etiologies of persistent eyelid inflammation were considered. The lesions were confined to the lower palpebral conjunctiva. Examination revealed clean upper and lower puncta without discharge from either punctum, making canaliculitis or dacryocystitis unlikely. As there was no clinical evidence of orbital or lacrimal drainage involvement, computed tomography, magnetic resonance imaging, or ultrasonographic imaging was not performed.

**Figure 2.**
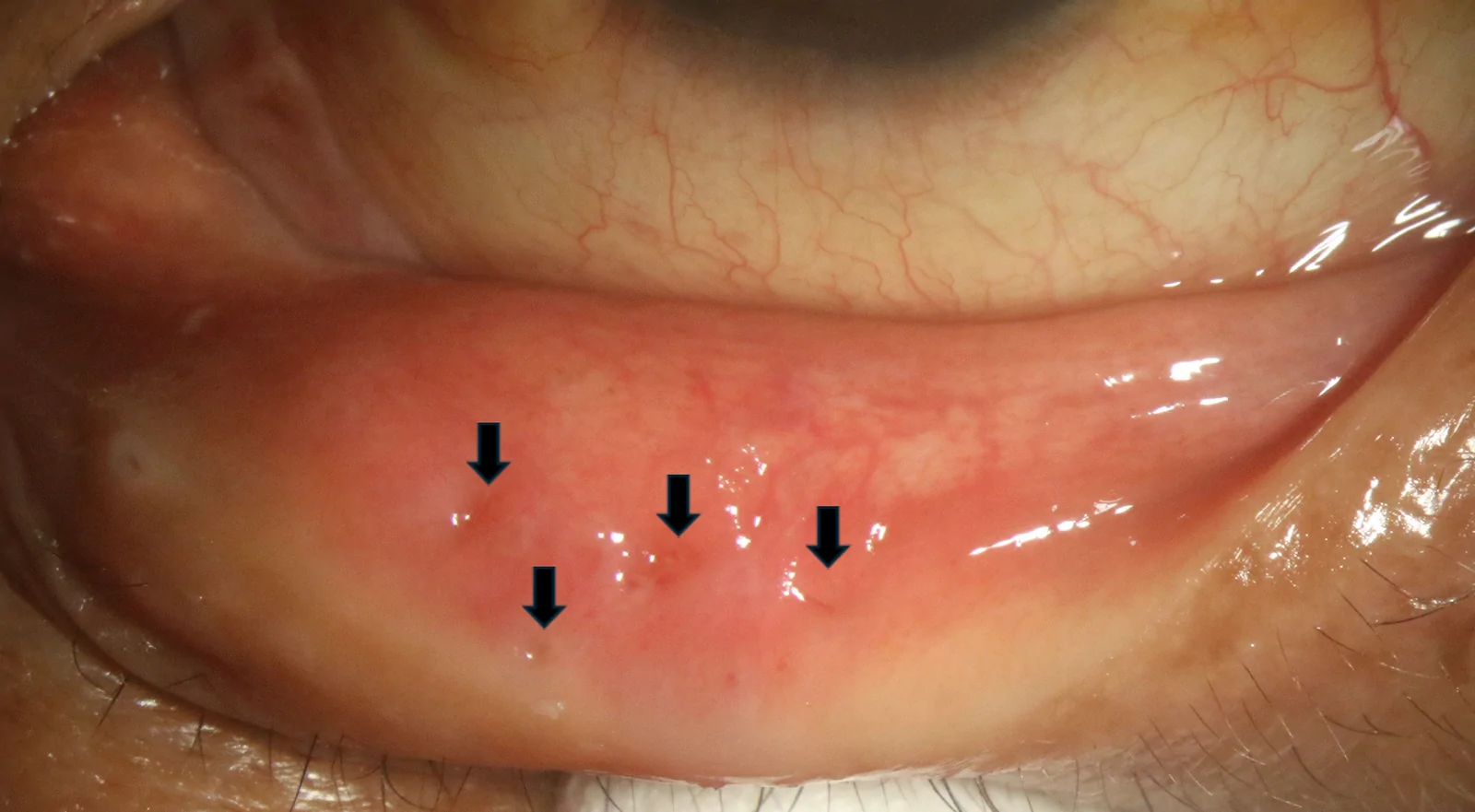
Clinical photograph of the left eye 2 years after the initial presentation showing persistent fistulous openings on the lower palpebral conjunctiva (arrows), accompanied by chronic eyelid thickening and induration.

Because of the chronic course of the infection and persistence of the fistulous lesions, surgical intervention was performed under local anesthesia and aseptic conditions. The involved tissue and associated fistulous tissue of the left lower palpebral conjunctiva were excised using a No. 15 blade, followed by limited debridement of visibly involved tissue and bipolar electrocautery as needed. Purulent material from the fistulae was collected for microbiological culture, while the excised tissue specimen was submitted separately for histopathological examination. After hemostasis was achieved using bipolar cautery, the conjunctival wound was closed with 3 interrupted 8-0 Vicryl sutures. Tobramycin/dexamethasone ophthalmic ointment was then applied, and the eye was patched. Postoperatively, topical ciprofloxacin was prescribed every 4 hours and tobramycin ophthalmic ointment twice daily for 2 weeks. Ciprofloxacin was continued every 4 hours for a total of 1 month. After the surgical wound remained stable without discharge, ciprofloxacin was reduced to once daily and continued for an additional 3 months. Surgical exploration revealed no foreign body or granulation tissue. Intraoperative cultures yielded MRSA and *M morganii*. Because the initial *S aureus* isolate was oxacillin-susceptible and molecular typing was not performed, the relationship between the initial and subsequent *S aureus* isolates could not be determined. Although the intraoperative culture subsequently yielded MRSA and *M morganii*, the MRSA isolate was not tested for ciprofloxacin susceptibility. Because the infected and fistulous tissue had been surgically excised and the postoperative wound showed progressive clinical improvement without persistent discharge or other evidence of ongoing infection, no additional antimicrobial therapy was initiated in response to the MRSA culture result. Histopathological examination revealed significant acute and chronic conjunctival inflammation with well-formed fistulous tracts (Fig. 3), consistent with a long-standing infectious and inflammatory process. No evidence of neoplasia was identified. Although atypical infections, including mycobacterial, fungal, actinomycotic, and tuberculous infections, were also considered in the differential diagnosis, specific microbiological testing for these pathogens was not performed.

**Figure 3.**
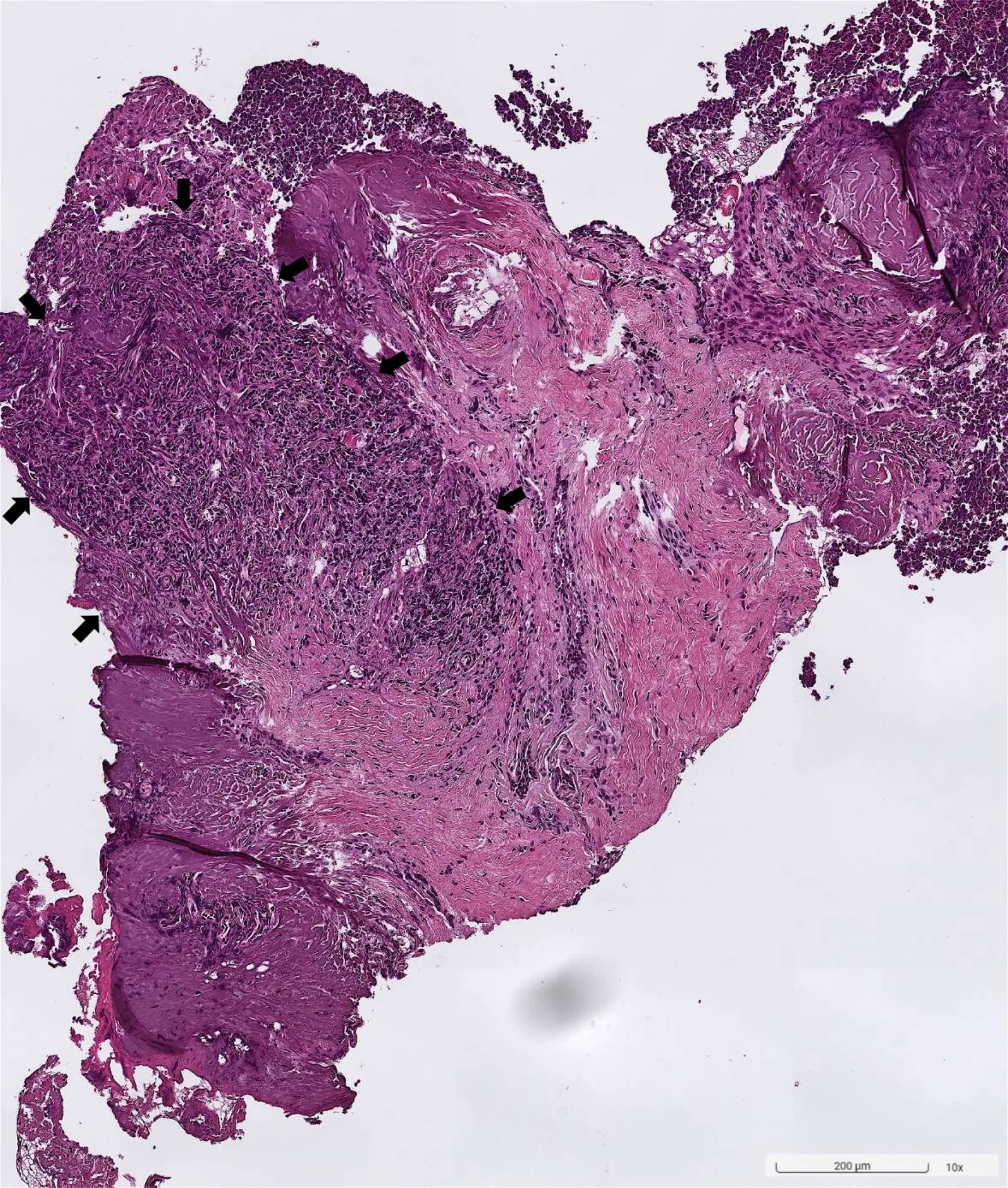
Histopathological examination of the excised conjunctival lesion (hematoxylin and eosin stain; 10 × objective, total magnification × 100) demonstrating dense acute and chronic inflammatory cell infiltration with fibrosis and fistulous tract formation (arrows). Focal cauterization artifact is present. No granulomatous inflammation or malignancy is identified on routine hematoxylin and eosin sections.

One month postoperatively, the eyelid mass had regressed substantially, the conjunctival fistulae had closed, and discharge had completely resolved (Fig. 4). The patient was followed for 1.5 years postoperatively. At the final follow-up, the eyelid remained stable, with no recurrence of fistulae or discharge, and the sensation of eyelid fullness had resolved. This case illustrates that chronic polymicrobial periocular infection may be associated with progressive structural changes, including fistula formation, and may require surgical management when prolonged medical therapy is insufficient.

**Figure 4.**
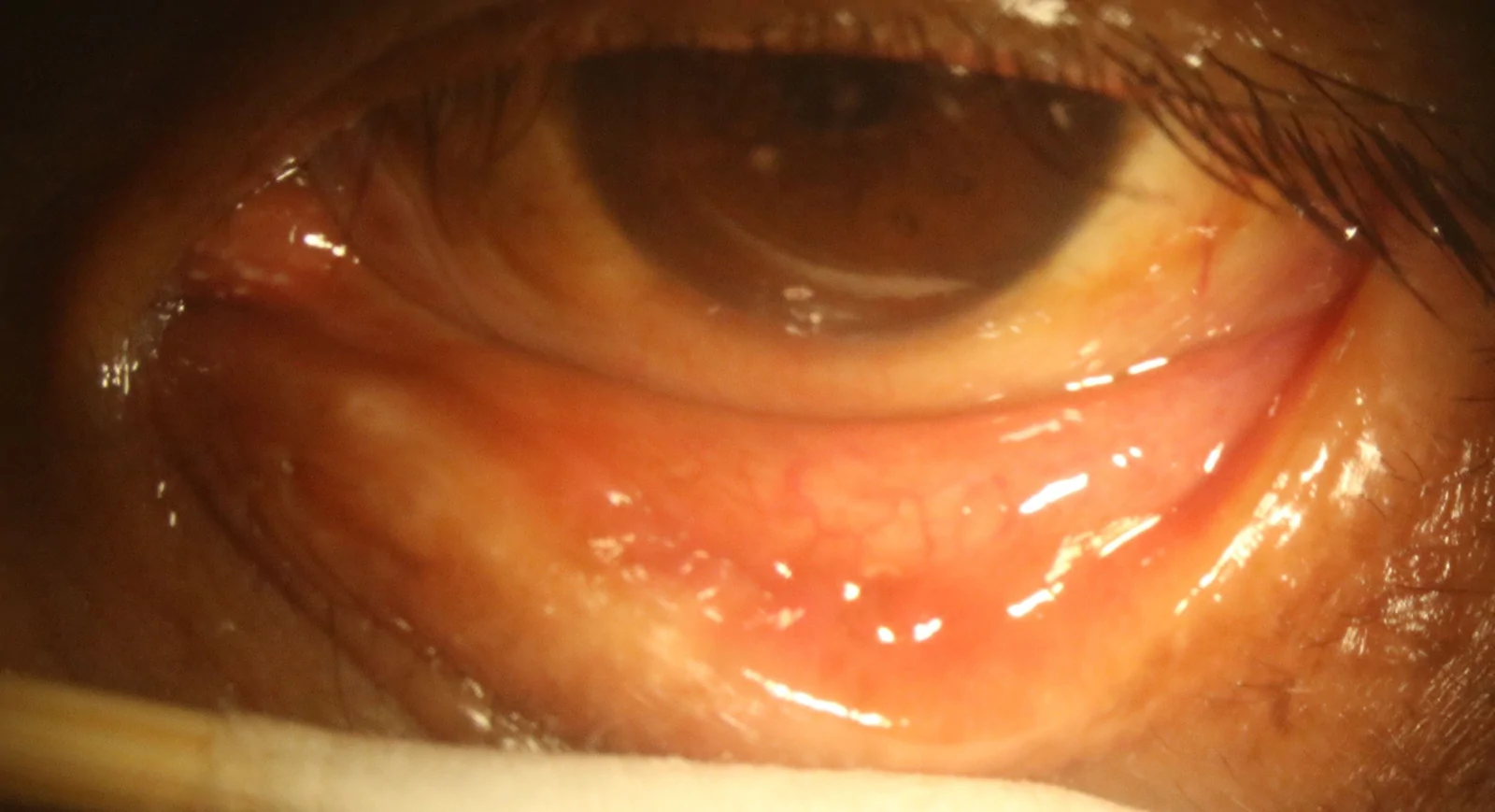
Clinical appearance 1 month after eyelid surgery, showing marked regression of the eyelid lesion, closure of the conjunctival fistulae, and resolution of discharge.

## 3. Discussion

This case describes an unusual presentation of chronic polymicrobial eyelid and palpebral conjunctival infection involving *S aureus* and *M morganii*, with MRSA isolated at the time of surgery. Although *M morganii* is a recognized cause of systemic infections, postoperative wounds, urinary tract disease, and bacteremia, ocular involvement is distinctly uncommon. Large prospective microbiological studies of bacterial keratitis have reported *M morganii* in approximately 1% of isolates,^[1]^ underscoring its rarity in ophthalmic practice. Even fewer cases of ocular adnexal disease have been documented, including orbital abscess secondary to dacryocystitis or periocular soft-tissue infection following trauma or immunocompromise.^[2]^ The isolation of *M morganii* in this chronic conjunctival and eyelid infection highlights its potential contribution to persistent disease in susceptible individuals.

Although glycemic control was generally adequate and the patient had no known immunodeficiency or history of recurrent infections, the combination of long-standing diabetes mellitus and end-stage renal disease requiring maintenance hemodialysis may have predisposed the patient to opportunistic infection and hindered resolution of the chronic infectious process. Long-term exposure to healthcare settings, including maintenance hemodialysis, may have also facilitated colonization or acquisition of multidrug-resistant organisms. These factors may have contributed to the prolonged clinical course observed in our patient.^[7]^

*M morganii* has attracted increasing clinical attention for its capacity to acquire antimicrobial-resistance determinants, including extended-spectrum β-lactamases and fluoroquinolone-resistance genes.^[3]^ The resistance profile of *M morganii* is of particular concern, as recent surveillance studies have documented increasing resistance among clinical isolates to multiple antimicrobial classes, including third-generation cephalosporins, fluoroquinolones, aminoglycosides, and even carbapenems. These findings emphasize the importance of culture-guided antimicrobial therapy and prudent antimicrobial stewardship.^[8]^ The chronic course may have been influenced by antimicrobial resistance and structural fistula formation. Although biofilm formation or sequestration of organisms within the fistulous tracts could theoretically have contributed to persistence, these mechanisms were not directly evaluated in this patient. The evolution of the discharge from yellow mucopurulent secretions to a thick, toothpaste-like material is consistent with a long-standing, partially contained infection characterized by intermittent epithelial breakdown and progressive sinus-tract development. Although the *M morganii* remained susceptible to levofloxacin, persistence of the infection despite prolonged antimicrobial treatment suggests that the established fistulous tracts and surrounding structural changes may have contributed to incomplete microbial eradication, although a protected microbial reservoir was not directly demonstrated.

The polymicrobial nature of this infection also merits consideration. Although *S aureus* commonly colonizes ocular tissues, recent studies have demonstrated substantial phenotypic diversity and frequent antimicrobial resistance among ocular staphylococcal isolates, even in healthy individuals. These observations indicate that the clinical significance of Staphylococcus isolation should be interpreted in conjunction with the clinical presentation and microbiological context rather than being regarded as evidence of pathogenicity alone. In our patient, the recovery of *S aureus* together with *M morganii* at both the initial presentation and the time of surgery, along with progressive fistula formation and histopathological evidence of chronic inflammation, supported the diagnosis of clinically significant polymicrobial infection rather than simple colonization.^[9]^

The identification of MRSA at the time of surgery may have contributed to the complexity of the clinical presentation. MRSA is a major pathogen in ocular and periocular infections and has been associated with severe manifestations, including preseptal cellulitis, lid abscesses, or orbital cellulitis.^[5]^ Several studies have shown that MRSA-related orbital infections may present atypically, often in the absence of the classic preceding history of sinusitis, with manifestations such as lacrimal gland involvement, insidious eyelid swelling, or multiple small orbital abscesses.^[4,5]^ Community-associated MRSA infections may behave aggressively and often require prompt surgical drainage in addition to antimicrobial therapy.^[6]^ The chronic structural changes observed in our patient (including multiple conjunctival openings and persistent fistulous tracts) differed from the overt tissue necrosis described in MRSA-associated necrotizing periocular infections.^[10]^ Nevertheless, the identification of MRSA at surgery may have contributed to the complexity of the polymicrobial infection.

Notably, the infection persisted for nearly 2 years despite prolonged intermittent topical antimicrobial therapy, including courses of vancomycin and fluoroquinolones. This suggests that while medical therapy may transiently suppress superficial infection, chronic structural changes of the conjunctiva and eyelid may contribute to bacterial persistence. Histopathological examination revealed established fistulous tracts accompanied by chronic inflammatory and fibrotic changes, indicating that long-standing structural remodeling had already occurred. In this patient, the presence of established fistulous tracts and surrounding fibrosis may have contributed to the persistence of infection despite antimicrobial therapy. However, sequestration of organisms within these tracts was not directly demonstrated. Although chronic conjunctival fistulae are rare, they may arise in the setting of long-standing inflammation, trauma, or abnormal tissue healing. In this patient, the combination of prior vitrectomy, diabetes, and end-stage renal disease may have increased susceptibility to persistent infection and impaired resolution.

Surgical intervention was undertaken after prolonged medical therapy failed to control infection, and progressive fistulae formation and eyelid induration persisted. Surgical excision and limited debridement facilitated removal of chronically infected tissue and restoration of anatomical continuity. The marked postoperative improvement characterized by closure of fistulae, resolution of discharge, and regression of eyelid thickening, underscores the role of surgery in cases where structural abnormalities perpetuate infection. Similar benefits have been demonstrated in complex periocular or orbital infections in which surgical drainage or debridement significantly enhanced outcomes, even in the presence of resistant organisms.^[4–6]^

Chronic eyelid or conjunctival infections involving gram-negative opportunistic pathogens are rarely described. Reported cases of *M morganii* infections have primarily involved trauma, envenomation, or extension from dacryocystitis.^[2,11]^ Severe periocular infections such as necrotizing fasciitis have likewise been associated with polymicrobial infections involving resistant staphylococci and streptococci, emphasizing the importance of early recognition and appropriate intervention.^[10]^ Although overt necrosis was not evident in the present case, prolonged inflammation was associated with substantial structural remodeling and fistula formation. Because the specimen was not evaluated with special histochemical stains and targeted microbiological testing, atypical bacterial, fungal, or mycobacterial infections could not be definitively excluded. However, bacterial cultures obtained at both time points yielded *S aureus* and *M morganii*, although the antimicrobial susceptibility profile of the *S aureus* isolate differed between the initial and intraoperative cultures, supporting the diagnosis of a chronic polymicrobial bacterial infection.

Importantly, this case highlights the value of repeat microbiological cultures and reassessment for structural abnormalities when eyelid or conjunctival infection persists despite apparently appropriate culture-directed antimicrobial therapy. Changes in antimicrobial susceptibility over time and the development of fistulous tracts may alter subsequent management. In susceptible hosts with underlying systemic comorbidities, persistent or recurrent discharge should prompt reconsideration of both the microbiological findings and the underlying tissue anatomy. Clinicians should maintain a high index of suspicion for unusual organisms, especially in patients with diabetes and end-stage renal disease or those with a history of ophthalmic surgeries.

In summary, we describe a chronic polymicrobial eyelid and conjunctival infection involving *S aureus* and *M morganii*, with MRSA isolated at the time of surgery. The infection followed a prolonged course with multiple conjunctival fistulae and significant eyelid induration. This case highlights the need to consider uncommon opportunistic pathogens in persistent periocular infections and demonstrates that antimicrobial therapy may be insufficient once structural tissue abnormalities and fistulous tracts become established. In this patient, surgical excision and limited debridement resulted in durable clinical resolution after medical treatment alone failed to resolve the chronic fistulae.

## 4. Conclusion

This case describes a chronic polymicrobial eyelid and conjunctival infection associated with *S aureus* and *M morganii*, with MRSA identified at the time of surgery. The prolonged clinical course likely reflected the interplay of polymicrobial infection, underlying systemic comorbidities, chronic tissue remodeling, and fistula formation. *M morganii* may have played a contributory role in sustaining chronic periocular infection in this susceptible patient, although it was unlikely to be the sole determinant of the prolonged disease course. In this patient, surgical excision and limited debridement achieved sustained resolution of the fistulae and discharge following the failure of extended medical treatment to resolve the underlying structural abnormalities.

## Acknowledgments

The authors thank the medical team at Keelung Chang Gung Memorial Hospital for their dedicated care of this patient and for their clinical support during case management. The authors also acknowledge the use of ChatGPT (OpenAI, GPT-5.5) as an artificial intelligence–assisted language tool for assistance in organizing figure legends and formatting citation styles. All clinical interpretations, data analysis, and final manuscript content were independently reviewed and approved by the authors.

## Author contributions

**Conceptualization:** Ju-Wen Yang.

**Data curation:** Ju-Wen Yang.

**Formal analysis:** Ju-Wen Yang, Liang-Che Chang.

**Methodology:** Liang-Che Chang.

**Supervision:** Liang-Che Chang.

**Visualization:** Liang-Che Chang.

**Writing – original draft:** Ju-Wen Yang.

**Writing – review & editing:** Ju-Wen Yang, Liang-Che Chang.
